# Dimensionality and Predictive validity of the Classroom Learning Activities Checklist in Prekindergarten

**DOI:** 10.1007/s11092-019-09306-7

**Published:** 2019-11-09

**Authors:** Arthur J. Reynolds, Allyson J. Candee

**Affiliations:** 1 Human Capital Research Collaborative and Institute of Child Development, University of Minnesota, 51 East River Road, Minneapolis, MN 55455 USA; 2 Institute for Community Integration, University of Minnesota, Minneapolis, USA

**Keywords:** Classroom assessment, Evaluation, Prekindergarten, School readiness, Predictive validity

## Abstract

The Classroom Learning Activities Checklist (CLAC) is a brief classroom observation measure that assesses task-oriented and self-regulated learning in early childhood environments. We assessed the tool’s dimensionality and validity in predicting prekindergarten (PreK) learning gains. The study sample is from the Midwest Child-Parent Center (MCPC) program, an evidence-based PreK–3rd grade school reform model providing comprehensive educational and family support services. Data from 1358 enrolled students in 72 observed classrooms indicated that a 2-factor model—instructional responsiveness and student engagement—explained 50% of the variance in item scores. Evidence for construct validity was strong. Linear and probit regression analyses indicated that CLAC scores independently predicted learning gains in literacy (ES = .34 *SD*) and math (ES = .30 *SD*) on the Teaching Strategies Gold Assessment System, a standardized performance assessment. Findings support the validity of the CLAC in assessing the classroom learning environment. Implications for program monitoring, evaluation, and professional development are discussed.

It has long been recognized that early childhood programs of high quality promote children’s school readiness and longer-term educational success (Camilli et al. 2010; Karoly et al. 2005). These identified gains have also been found to lead to economic and social benefits in adulthood (Cannon et al. 2017; Reynolds and Temple 2008). Since 4 in 5 children in the USA between the ages of 3 and 5 years old participate in center-based education and care for at least part of the day (U. S. Department of Education 2017), ensuring that programs are high quality is a major priority. However, wide variability in quality is the norm, especially for children from low-income families.

Defining the “high quality” in early childhood classrooms is challenging because it relies on the assumption that there is (a) consensus as what children should know and do at kindergarten entrance while (b) being able to meaningfully identify, measure, and support these behaviors and skills. Quality is typically defined by either structural indicators (e.g., group sizes, teacher-child ratios, teacher qualifications; Vandell and Wolfe 2000) or process indicators (e.g., teacher-child interactions; Phillipsen et al. 1997).

In center-/classroom-based early childhood learning environments, specific aspects of classroom practice have been linked to children’s social and academic outcomes. For example, teachers’ levels of education (minimum bachelor’s degree) are associated with children’s development of social competence (Mashburn et al. 2008) and higher receptive language skills (Burchinal, Cryer, Clifford, & Howes, 2002). Further, high-quality instructional practices and teacher-child interactions in early childhood programming have replicated linkages to children’s academic and social development (Gosse et al. 2014; Mashburn et al. 2008; Wasik and Hindman 2014). Both quality factors are important in supporting children’s gains, yet structural features may be necessary for establishing a supportive context for learning. Below we summarize key learning experiences that are promoted by high quality classroom environments and their relations with school achievement.

## Defining children’s learning experiences

Another approach for defining and categorizing classroom quality is a two-part process: (1) identify child-level characteristics and skills that predict later learning and then (2) isolate specific classroom practices, characteristics, and environments that support them. Both children’s academic and nonacademic skills predict later school success. While it is clear that early literacy and number skills predict later learning in many domains (Reynolds 2000, 2019; Storch and Whitehurst 2002; Wasik and Hindman 2011), a separate set of self-regulatory skills may independently contribute to children’s achievement and well-being. Evidence suggests self-regulation development via self-directed learning and classroom engagement uniquely contributes to and fosters children’s learning (e.g., Fantuzzo et al. 2004; Fitzpatrick and Pagani 2013).

### Self-regulation

Self-regulation is a broad construct that can be loosely defined as sets of skills that promote the development of children’s purposeful control of thoughts and actions (Blair et al. 2005). Self-regulatory skills form a critical foundation that undergirds children’s cognitive and socio-emotional development. A balance between emotional physiology and cognitive regulation emerges from self-regulation. Children’s goal-oriented learning is promoted through the management and control of attention and emotion.

Children’s early self-regulation and their approach to learning have been linked to a wide range of outcomes, including literacy skill development and early math skills (Blair and Razza 2007; Dobbs-Oates et al. 2011), positive adaptive behavior and reductions to problem behavior (Rimm-Kaufman et al. 2009), and later achievement (Duncan et al. 2007). When translating self-regulatory behaviors into classroom skills that connect to later learning, specific behaviors of engagement, task orientation, effortful attention, and emotional regulation can be identified and isolated (Blair and Raver 2014).

### Task orientation

Task orientation is a classroom skill where children actively facilitate their learning via direct and focused attention to the learning activities. It is operationalized as active engagement in learning that is necessary to successfully navigate through a series of tasks to reach a learning goal. Historically, task orientation has been a key dimension of classroom learning environments (Fraser 1998). Active participation and involvement help define the social climate of the classroom (Moos 1979). Based on Murray’s (1938) concept of environmental “press,” the Classroom Environment Scale (Moos and Trickett 1974), for example, measures perceptions of the extent to which the setting emphasizes task orientation, involvement, affiliation, and teacher support. Further, students regulate their emotions and reactions to effectively direct attention to the current task. Self-control and self-directed learning are thus critical in task orientation: students must effectively focus attention and regulate behavior to match the learning activities in the classroom. Task orientation, whether assessed individually or as a dimension of the environment, uniquely contributes to later school performance, achievement, and social functioning (Hightower 1986; Kohn and Rosman 1973; Moos and Moos 1978).

## Classroom characteristics

Certain classroom features theoretically promote or, at a minimum, moderate students’ task orientation and engagement in the classroom. A balanced instruction model of teacher-directed and child-initiated learning and the presence and absence of student misbehavior are characteristics that potentially facilitate or, conversely, impede task-oriented learning.

### Instructional balance

There is evidence that a balanced approach of teacher-directed and child-initiated learning promotes achievement (Graue et al. 2004). Early childhood instructional approaches can be categorized as those that emphasize either teacher-directed or child-initiated learning. The driver defines the distinction between the two approaches: In teacher-directed programming, learning is organized and sequenced by the teacher where child-initiated activities and environments may be planned by the teacher but chosen by the student. A balanced approach, defined as a nearly equal percentage of instructional time between the two types, promotes active learning that leads to greater content mastery. Conceivably, classrooms that employ a blended instructional approach will simultaneously have higher levels of task orientation. Classrooms with a balance of teacher-directed and child-initiated instruction are associated with greater gains in school readiness, reading and math achievement in the elementary grades, and higher rates of school completion (Graue et al. 2004; Reynolds 2000; Clements et al., 2004)

### Student behavior

Student behavior in the classroom is affected by and in turn affects task orientation. First, an individual student’s self-control and ability to follow classroom expectations may promote (or alternately inhibit) the individual learner while simultaneously affecting the overall classroom environment (e.g., disrupting others’ attentive learning vs. demonstrating positive learning behaviors). Secondly, the classroom environment may promote task-oriented behaviors through effective classroom management strategies and clear and consistent behavioral expectations. Classroom-wide behavior management supports can reduce preschoolers’ externalizing and internalizing problems (Han et al. 2005) and promote appropriate student behavior (Hiralall and Martens 1998).

To date, no process quality assessments in early childhood specifically focus on task orientation (e.g., task persistence, attentiveness, self-directedness, active engagement). While other classroom tools, e.g., the Classroom Assessment and Scoring System (CLASS; Pianta et al. 2008) and Early Childhood Environment Rating Scale-Revised (ECERS-R; Harms et al. 2005), have highlighted aspects of these behaviors, they often aggregate specific task-oriented behaviors into larger constructs (e.g., organization, emotional support), prohibiting the ability to assess the behaviors’ unique contribution. Moreover, given the extensive resources necessary to both train and assess classrooms using the available classroom instruments, a significant need in the early childhood field is a brief tool that measures the unique classroom behaviors that support students’ self-regulation and task orientation.

## Research Questions

This study assesses the psychometric characteristics of the Classroom Learning Activities Checklist (CLAC) by answering two main questions:

1. What is the dimensionality of the CLAC observational assessment?
2. Is there evidence of predictive validity? To what degree does CLAC predict children’s learning at the end of preschool in multiple domains of school readiness?

## Methods

### Sample and setting

Study participants are part of the Midwest Child-Parent Center (MCPC) Expansion Project, an evidence-based prekindergarten (PreK)-3rd grade school reform model implemented beginning in 2012–2013 in four school districts (Reynolds et al. 2014, Reynolds et al. 2016a, b). In order of size, they are the Chicago Public Schools, Saint Paul Public Schools, Evanston-Skokie District 65, and McLean County Unit District 5. MCPC is funded by an Investing in Innovation (i3) grant from the U.S. Department of Education. The 5-year intervention provides comprehensive family and school support services to a cohort of children from PreK to 3rd grade (Reynolds et al. 2016a, b, 2017). The six core elements are collaborative leadership, effective learning experiences, aligned curriculum, professional development, parent involvement and engagement, and continuity and stability. School, classroom, and teacher services include an aligned professional development/coaching model, leadership support, classroom aides, and vertically and horizontally aligned curricula. A total of 98% of the PreK teachers had at least Bachelor’s degrees with an average of eight years of teaching experience.

As shown in Table 1, the original sample is a PreK cohort of 3535 students in 46 schools (2323 program and 1212 comparison-group children). The comparison group enrolled in the usual district preschool programs in schools matched on propensity scores (student demographic characteristics and 3rd grade test scores). Although the present study does not assess the intervention, this school context provides a description of the sample selection.

**Table 1:** Midwest Child-Parent Center (MCPC) school, classroom, and student sample sizes

School and classroom context	Original study sample	CLAC study sample	Validity sample (Chicago)	% 4-year-olds
Number of schools	46	33	24	–
Program, comparison	25, 21	25, 8	16, 8	–
Number of classrooms	116	72	54	–
Program, comparison	88, 28	64, 8	46, 8	–
Number of students	3535	2232	1358	70%, 70%
Program, comparison	2323, 1212	1950, 282	1134, 224	68%, 81%
Chicago schools	30	24	24	–
Classrooms and students	86, 2630	54, 1358	54, 1358	60%, 61%
Outside Chicago schools	16	9	N/A	–
Classrooms and students	30, 905	18, 874	N/A	89%, 85%

Two schools were affiliated child care centers. The Virginia (MN) site (53 children in 3 classrooms) was dropped from the project mid-year and is excluded. The Evanston comparison group was in the same school as CPC. CLAC sample was a random selection of one or two classrooms in each program site and in 8 Chicago comparison sites. % 4-year-olds are provided for CLAC and validity study samples

Two samples for the current study were defined. The CLAC study sample included 72 (out of 116) randomly selected classrooms from program (*n* = 64) and comparison schools (*n* = 8; Chicago only). One or two classrooms were selected from each school depending on the number of PreK rooms. Accounting for morning and afternoon sessions in most classrooms, this included a total of 2232 students (see Table 1). Because Chicago is over 70% of the total sample, the validity sample was restricted to this district. Included were 54 classrooms (24 schools) with 1358 enrolled students, 60% of whom were 4-year-olds. All districts schools use the Teaching Strategies Gold Assessment System (TS-Gold; Heroman et al. 2010), a standardized performance assessment of school readiness skills. Table 2 shows the demographic characteristics of the validity sample.

**Table 2:** Student characteristic and covariate sample sizes, means, standard deviations, and response ranges

Student variable^a^	*M*	(*SD*)	Range
Gender (Female)	.52	(0.50)	0–1
Black (African-American)	.68	(0.47)	0–1
Hispanic	.31	(0.46)	0–1
Special education placement	.07	(0.26)	0–1
Age in months	48.25	(6.48)	35.35–58.84
Eligible for subsidized lunch program	.86	(0.35)	0–1
Fall assessment was after October (1 = yes)	.41	(.49)	0–1
Baseline TS-Gold math skills	22.44	(8.63)	0–56
Baseline TS-Gold literacy	33.35	(15.50)	0–92
Baseline TS-Gold socio-emotional	39.91	(12.86)	0–81
Baseline TS-Gold language skills	28.16	(7.76)	1.5–54
Baseline total score (all subscales)	190.53	(58.77)	10.45–386

^a^*n* = 1358

*TS-Gold* Teaching Strategies Gold Assessment System, a standardized performance assessment. The means of dichotomous variables are proportions indicating the percentage of students in the respective category.

### Classroom Learning Activities Checklist

As part of the study, classroom observations were conducted using the Classroom Learning Activities Checklist (CLAC), an internally created assessment that captured the nature and quality of student task orientation and the classroom practices that support it. Roughly one-half of the prekindergarten classrooms in each of the implementation sites and one classroom from each control site were randomly sampled.

The assessment tool was designed to be consistent with principles of effective learning environments described in the introduction and included content on engaged instruction and self-regulation, an enriching classroom climate, task-oriented goals and experiences, and active learning and child-initiated activities (Graue et al. 2004). These principles and foci are key elements in the CPC program and other effective interventions leading to beneficial long-term effects (Ramey and Ramey 1998; Reynolds et al. 2017; Reynolds and Temple 2019).

The CLAC is organized into 4 theoretically constructed domains: (a) items one through six inquire about observed student task-oriented behaviors; (b) items seven through 17 measure the provision and facilitation of learning activities that support task orientation; (c) items 18a–c, 19, and 20 assess how instructional time is spent; and (d) items 21–23 measure the presence and absence of student misbehavior. Each of these items is coded on a 1–5 Likert scale (1 = strongly disagree/never/none, 2 = disagree/rarely/few, 3 = neutral/sometimes/some, 4 = agree/most of the time/many, 5 = strongly agrees/always/nearly all) and has descriptions of each of the scores in a scoring rubric. Finally, item 26 (CLAC26) rates the overall level of task orientation in the classrooms. Assessors incorporate the four constructs into a single 1–5 score: 1 = very low, 2 = moderately low, 3 = somewhat, 4 = moderately high, 5 = very high. See Table 4 for a list of CLAC items 1–23 and item 26 (Table 9 for a complete list).

#### Other recorded information

Additional information was collected on the CLAC tool: number of assistants, observation start and end times, number of children present, description of activities (open- ended), content focus (art, fine motor, language/literacy, math/number concepts, science, socio- emotional), group organization (whole group, small group, individual time, free choice, and routines), age ranges (3’s, 4’s, or mix), program length, and curricula used.

### Procedures

#### Training and reliability

Trained observers conducted all of the CLAC observations. Prior to conducting the CLAC, observers participated in a 6-h training where constructs/subscales, items, and the general purpose of the tool were described. Observers learned about the organization of the tool and viewed subscale- and item-level video clips. Scoring guidelines and observation protocols were presented, and finally, participants jointly practiced coding video clips using the CLAC**.**

Reliability was established by assessors independently viewing and coding two 20-min online PreK classroom videos. The videos were intentionally selected so that a range of low-, mid-, and high-range behaviors was presented. Additionally, both whole group instruction and free choice groupings were selected to assess observers’ ability to reliably code behaviors in different classroom structures. For the purposes of training and quality assurance, each item was scored for each video and master scores were created through consensus from two of the tool authors. These scores were used to gauge observers’ knowledge of the CLAC’s content and scoring structure. A small number of dual-coded field observations were also conducted throughout the observation window. Inter-rater reliability was estimated for these observations and found to be in the acceptable range.

#### Observation process

In each observed classroom, trained assessors conducted a 25–30-min classroom observation. This length was selected because (1) it ideally permitted more than one group setting in PreK classrooms while (2) remaining a brief snapshot of a typical day. There was an approximate 1-week window from the initial CLAC training and reliability testing to data collection in the field. At the end of the observation, the assessor referred to a complementary matrix for item-level descriptions and response ranges and coded each item on the 5-point Likert scale. The ratings were derived from extensive notes made during the observations.

### Measures

#### Outcome measures

Student achievement was assessed using TS-Gold, a standardized teacher-reported performance assessment of school readiness skills. In the MCPC project, this observation assessment tool was administered in the fall (October–November) and the end of the prekindergarten year (mid-May) by classroom teachers. The following subscales with their summed individual items (after adjusting for age) were used in this study: oral language (6 items), literacy (12 items), social-emotional (9 items), and mathematics/numeracy (7 items). Each item was given a score from 0 (not yet meeting objective) to 9 (full mastery of objective). A total score was comprised of all raw scores in all domains. Additionally, proficiency variables were created using performance at or above national norms where 1 = met national norm on 4 or more subscales and 0 = less than 4 were met (Lambert et al. 2014).

Item reliabilities are .99 for each of the six scales; person reliabilities and internal consistency estimates range from .95 to .98 and .96 to .98, respectively. Inter-rater reliability was at or above .80 (Reynolds et al., 2014, 2016b). Additionally, TS-Gold scores highly correlate with other direct assessments (Lambert et al. 2014) and may be used with diverse learners and populations—differential item analysis indicates the measure demonstrates validity evidence for children with special needs and English language learners (Lambert et al., 2014; Reynolds et al., 2014 [Online supplement])).

#### Covariates

To account for individual, family, and school/neighborhood differences, a comprehensive set of control variables was included in predictive validity evidence analyses. Previous learning was controlled by using a continuous measure of fall performance: the baseline variable matched the outcome variable (e.g., baseline literacy score was used for literacy outcomes). To account for learning/maturation effects due to the date of assessment, an assessment date variable was included as a control and was defined as follows: 1 = assessment completed prior to October and 0 = after October. Race was dichotomously coded as 1 = African-American and 0 = other. Ethnicity was coded 1 = Hispanic and 0 = non-Hispanic. Gender was dichotomized (1 = female; 0 = male). Special education status was defined as 1 = yes and 0 = no. Age was coded as the students’ age in months at kindergarten entry. Free and reduced lunch status was used as an indicator of socio-economic status: 1 = free lunch eligible and 0 = not eligible. See Table 2 for the means, standard deviations, and response ranges for the covariates included in the regression models.

### Analyses

A series of descriptive statistics were examined to explore the dimensionality of the CLAC. Exploratory factor analysis (principal components analysis) was conducted using SPSS version 14 and was used to (a) identify the dimensionality of the task orientation via reviewing the variance structure and (b) create factor scores that describe characteristics of task orientation for later regression analyses. The variables constructed from factor analysis were used in later predictive validity evidence analyses. Given that the CLAC tool was newly developed to measure task orientation and related attributes of quality, an exploratory analysis to identify the number of dimension using principal components analysis was preferred over a confirmatory factor analytic approach.

To assess the validity of CLAC scores in predicting later children’s learning, probit and multiple linear regression were used using STATA version 13. Linear regression analyses measured the relation of CLAC predictor variables to continuous outcome variables—children’s TS-Gold scores in language, literacy, math, socio-emotional, and total sum scores. To capture the potential impact on a minimum threshold of necessary learning, probit regression analyzes dichotomized outcomes of children’s proficiency where 1 = met national scores and 0 = did not meet nationally normed averages. Similarly, the relations among the CLAC variables with covariates to these binary scores were used to predict children’s language, literacy, math, and socio-emotional proficiency scores.

Regression coefficients in each model were used as indicators of the strength of relation between predictor variables (or covariate) and the outcome measure. Coefficients, either negative or positive, with a *p* value below .05 were considered significantly associated with TS-Gold.

Instead of removing incomplete observations (where one or two time points were missing) and decreasing power from lower sample sizes (Nakagawa and Freckleton 2008), regression models were analyzed using an imputed dataset. Multiple imputation of missing data using an EM algorithm was used to generate maximum likelihood estimates. This imputation method is often considered superior to other procedures that handle missing data (Buhi et al. 2008; Cox et al. 2014) while maximizing the available sample. EM algorithms provide excellent parameter estimates that are close to the population average (Graham 2009). Outcome and demographic variables, including fall baseline performance scores, assessment date, age, race, special education status, free lunch eligibility, gender, proficiency in three or more domains, and a school-level reading achievement, were included in the algorithm to produce missing case parameter estimates (means, variances, and co-variances).

## Results

The purpose of this study was to explore the psychometric properties of the Classroom Learning Activities Checklist, including its internal design and structure, reliability, and validity evidence.

### Research question 1: what is the construct validity evidence and dimensionality of the tool?

#### Classroom observation features

The average CLAC observation was conducted in 31 min (*M* = 30.6, *SD* = 5.2) with 1.3 support staff members (in addition to the lead teacher) (*M* = 1.3, *SD* = .67) and 15 children (*M* = 14.6, *SD* =3.1). Nearly all of the observations were conducted in the morning, excluding classrooms that offered one-half day programming in the afternoon. At the beginning of the school year, the classroom enrolled either (a) exclusively 3-year-olds (8.3% of all observations), (b) exclusively 4-year-olds (33%), or (c) a mix of 3- and 4-year-olds (40.3%). See Table 3 for an overview of classroom characteristics.

**Table 3:** Classroom characteristics’ sample size, mean, standard deviation, and response range

Variable	*N*/frequency^a^	*M*/percent	*SD*	Range
CLAC observation length (min)	66	30.55	5.2	20–45
Number of support staff	69	1.29	0.67	0–3
Number of children	70	14.57	3.06	5–20
Classroom ages				
Classrooms with 3-year-olds	6	8.3		
Classrooms with 4-year-olds	24	33.3		
Mixed classrooms of 3s and 4s	29	40.3		
Primary content area				
Fine motor	1	1.4		
Language/literacy	25	34.7		
Math	1	1.4		
Science	3	4.2		
Socio-emotional	4	5.6		
Observed content area (1 = yes; 0 = no)				
Art	67	0.12	0.33	0–1
Fine motor	67	0.19	0.40	0–1
Language/literacy	67	0.85	0.36	0–1
Math	67	0.27	0.45	0–1
Science	67	0.28	0.45	0–1
Socio-emotional	67	0.21	0.41	0–1
Primary grouping				
Whole group	37	51.4		
Small group	2	2.8		
Free choice	9	12.5		
Observed grouping (1 = yes; 0 = no)				
Whole group	67	0.72	0.45	0–1
Small group	67	0.27	0.45	0–1
Free choice	67	0.39	0.49	0–1
Individual time	67	0.09	0.29	0–1
Routines	67	0.03	0.17	0–1

^a^Reported values from CLAC observations

#### Content topics

As seen in Table 3, the observed learning areas varied but language/literacy activities were consistently present (in 81% of all observations). Science and math activities were present in roughly the same number of observations (28% and 27%, respectively). Finally, fine motor and socio-emotional learning were observed in 19% and 21% of the classroom observations, respectively. CLAC observers also indicated the primary content area: language/literacy learning was the dominant content area in 73% of all observations.

#### Grouping

Whole group instruction was often observed (72% of the time). Free choice was offered in 39% of all observations; small group instruction was present in 27% of observations. Across CLAC observations, routines (e.g., meals, transitions) were noted 3% of the time. Whole group instruction was recorded as the primary grouping in over 77% of the CLAC observations.

#### CLAC dimensionality

See Table 4 for a complete list of the sample sizes, means, standard deviations, and response ranges for items 1–23, 26. There were between 66 and 72 cases for each of the items. The missing data appears to be random: in examining the paper copy observations, missing values were across different observations and different observers. Item averages, correlations, and factor scores used all available item-level scores. For factor analysis and regression models, missing observation scores were imputed by inserting site-level averages.

**Table 4:** Classroom Learning Activities Checklist (CLAC) items sample sizes, means, standard deviations, and response ranges

Variable	Number	*M*	*SD*	Skew	Kurtosis	Range
CLAC 1. Fully engaged in activities	72	4.31	0.68	− 0.73	3.44	2–5
CLAC 2. Active participants in learning	70	4.26	0.72	− 0.66	3.06	2–5
CLAC 3. Oriented to learning objective	72	4.31	0.68	− 0.47	2.19	3–5
CLAC 4. Engaged with peers and/or materials	72	4.11	0.83	− 0.65	2.81	2–5
CLAC 5. Attention to the lesson is evident	70	4.27	0.74	− 0.69	2.87	2–5
CLAC 6. Sharing of answers and thoughts	70	3.94	0.92	− 1.03	3.95	1–5
CLAC 7. Org. of lesson promotes task orien.	70	4.26	0.83	− 1.28	5.24	1–5
CLAC 8. Methods promote engagement	70	4.04	0.75	− 0.48	3.01	2–5
CLAC 9. Methods facilitate active part.	66	3.95	0.81	− 0.61	3.11	2–5
CLAC 10. Teacher open to active part. and eng.	68	4.16	0.78	− 0.66	2.97	2–5
CLAC 11. Individual attention to children	71	4.15	0.69	− 0.47	3.13	2–5
CLAC 12. Extra help is provided	68	4.06	0.73	− 0.32	2.62	2–5
CLAC 13. Responsiveness to work and behave	70	4.13	0.74	− 0.42	2.60	2–5
CLAC 14. Activities engage children	72	3.99	0.74	− 0.40	2.95	2–5
CLAC 15. Activities support active part	70	4.16	0.83	− 1.22	5.26	1–5
CLAC 16. A variety of activities are provided.	69	3.99	1.12	− 0.86	2.83	1–5
CLAC 17. Teacher- and child-directed activities	71	3.11	1.14	− 0.11	2.21	1–5
CLAC 18a. Time is lost due to lack of prep	70	4.90	0.35	− 3.65	16.58	3–5
CLAC 18b. Time is lost due to misbehavior	72	4.61	0.64	− 1.39	3.72	3–5
CLAC 18c. Time is lost due to routines	70	4.69	0.65	− 2.15	7.22	2–5
CLAC 19. Pace of activities matches interests	72	4.07	0.83	− 0.73	3.15	2–5
CLAC 20. Time in lessons matches interests	72	3.99	0.76	− 0.56	3.26	2–5
CLAC 21. Misbehavior is a problem	72	4.44	0.85	− 1.40	3.95	2–5
CLAC 22. Children follow rules and directions.	70	4.29	0.82	− 1.36	5.60	1–5
CLAC 23. Positive peer relations present	70	4.33	0.68	− 0.78	3.64	2–5
CLAC 26. Overall score	72	3.83	0.69	− 0.80	4.10	2–5

Overall, CLAC items scores were above the scale mean—all but one were above 3.83 on a 5-point Likert scale. As indicated by the means, all items were negatively skewed. Several other items (items 7, 15, 18b, 18c, 21, and 22) were negatively skewed between − 1.22 and − 2.15, suggesting the distributions were not normally dispersed. Item 17 (Learning time was lost due to teacher unpreparedness) appeared particularly problematic: the standard deviation was small (*SD* = .35) with a negatively skewed (− 3.65) and leptokurtic distribution (16.58).

In subsequent analyses, items 18a, 18b, 18c, and 21 were reverse coded to reflect the positive coding schema of the other CLAC items. These items (e.g., item 21 “Child misbehavior is a problem in this class”) were negatively worded, and changing the scoring structure allowed for easier interpretation across items. Once the reverse-coded items were converted, they also reflected the overall scoring trend of the other CLAC items.

#### Factor analysis

Factor analysis, a statistical technique of data reduction, was used to better capture the dimensionality of the CLAC. The smallest number of interpretable factors then represented latent constructs within the tool. Factor analyses were conducted using 25 CLAC items, including items 1–17, 18a–c, and 19–23.

#### Factor analysis process

The factor analysis process included two major questions: (a) if any, how many factors best describe the CLAC tool? and (b) What do they represent? Four criteria were used to determine factor retention: (a) using factors with an eigenvalue of least one, (b) visually determining the leveling off point (elbow) of the slope curve on the Scree plot, (c) adding factors until the total variance no longer increases substantially, and (d) ensuring each factor had more than three items as unique contributors.

The Kaiser-Meyer-Olkin Measure of Sampling Adequacy (KMO) analysis indicated our data was factor analyzable, given the output score was .81, well above the recommended minimum threshold of .5 (Kaiser 1970). For the extraction method, the models used principal component analysis (PCA). An oblique extraction method (direct Oblimin) was used.

Item loadings were defined as follows: 0–.2 = negligible/to be dropped, .3 = retained but not used as a factor indicator, and .4+ = factor contributors. Items were considered uniquely loading on one factor if the value was above .4 on one factor and below .3 on the others. Those items with factor loadings between .3 and .4 on multiple factors were counted as loading on each factor.

#### Results

In assessing the first criteria, there were six factors with eigenvalues above a value of one, as seen in Table 5. The rest of the factor criteria, however, indicated a 2-factor solution a better fit for the data. The Scree plot suggested the leveling off point between two and three factors, indicating a two-factor model better described the data. The cumulative variance was more difficult to interpret. The first factor explained 39% of the variance; a second factor added approximately 10%. For social sciences and humanities, the common variance can be as low as 50–60% (Hair et al. 2006). See Table 10 for CLAC factor loadings using orthogonal Varimax rotation.

**Table 5:** Percentage of total variance explained in factor analysis

Component	Initial eigenvalues			Extraction sums of squared loadings			Rotation sums of squared loadings^a^
	Total	% of Variance	Cumulative %	Total	% of Variance	Cumulative %	Total
1	9.84	39.36	39.36	9.84	39.36	39.36	6.51
2	2.60	10.38	49.75	2.56	10.38	49.75	4.71
3	1.73	6.91	56.66	1.73	6.91	56.66	2.46
4	1.42	5.67	62.33	1.42	5.67	62.33	5.31
5	1.21	4.83	67.16	1.21	4.83	67.16	5.57
6	1.03	4.13	71.29	1.03	4.13	71.29	1.91
7	0.82	3.27	74.56				
8	0.75	3.01	77.58				
9	0.74	2.97	80.54				
10	0.65	2.59	83.14				
11	0.63	2.52	85.65				
12	0.51	2.05	87.70				
13	0.47	1.89	89.59				
14	0.45	1.78	91.37				
15	0.37	1.49	92.86				
16	0.31	1.24	94.10				
17	0.29	1.14	95.25				
18	0.25	1.00	96.25				
19	0.21	0.82	97.07				
20	0.17	0.70	97.76				
21	0.15	0.60	98.36				
22	0.12	0.49	98.84				
23	0.12	0.47	99.32				
24	0.10	0.38	99.70				
25	0.08	0.31	100.00				

Extraction method: principal component analysis

^a^When components are correlated, sums of squared loadings cannot be added to obtain a total variance

Overall, results indicate a two-factor model best described the data, given the Scree plot and lack of higher-magnitude and distinctive item loadings on the 3rd–6th factors. Factor 1, labeled “instructional responsiveness,” was largely defined through observed teacher behaviors and the establishment of classroom practices that promote task orientation. Items included teachers’ responsiveness, assistance, and attention to individual students, teaching methods promoting engagement and active participation, provision of activities that promote active engagement and a balanced instructional approach, and pace and timing of lessons.

The items in the second factor, labeled “student engagement,” were student-observed behaviors and included observed engagement, students’ focus on learning activities, student behavior, and peer relations. Several of the items that loaded student engagement reflected observed child behavior (e.g., children follow rules, pay attention to lesson, classroom levels of misbehavior, and subsequent time lost due to misbehavior).

Three items loaded on both factors: items 2, 7, and 14. It is unclear if the items theoretically loaded on both factors or there are internal issues with the items. Finally, CLAC items 18a (Time lost to a lack of teacher preparedness) and 18c (Time lost in routines) were included but did not load on either factor above the .3 criteria. See Table 6 for pattern matrix with factor loadings for the 2-factor model.

**Table 6:** Classroom Learning Activities Checklist (CLAC) principle component analysis pattern matrix

	Component	
	1	2
15. Activities support active participation.	*.85*	− .04
16. A variety of activities are provided.	*.81*	− .09
8. Teaching methods promote engagement.	*.79*	.06
9. Teaching methods facilitate active participation.	*.77*	−.002
20. The amount of time in the lessons/activities matches children’s interests.	*.76*	.03
17. There is a blend of teacher-directed and child-initiated activities.	*.73*	− .18
11. Individual attention to children is evident.	*.69*	− .11
12. Extra help is provided to children when needed.	*.69*	− .18
10. Teacher shows openness/responsiveness to active part. and engagement.	*.67*	.07
6. Child sharing of answers and thoughts is frequently observed.	*.65*	.11
19. The pace of activities matches children’s interests and attention.	*.63*	.20
14. Activities provided consistently engage children.	*.55*	.37
4. Children are engaged with peers and/or materials.	*.53*	.28
13. Responsiveness to children’s work and behavior is frequent.	*.53*	.06
7. Organization of lesson and materials are conducive to task orientation.	*.50*	.33
2. Children are active participants in their learning.	*.49*	.35
18a. Time is lost to lack of teacher preparation. (reversed)	.21	.04
18c. Time is lost due to routines. (reversed)	.08	.001
23. Children demonstrate positive peer relations.	− .17	*.83*
22. Children follow rules and directions.	− .20	*.80*
1. Children appear fully engaged in activities.	.25	*.73*
3. Children appear to be working/oriented towards a goal/learning objective.	.19	*.70*
5. Children’s attention to the lesson is evident.	.23	*.68*
21. Misbehavior is a problem in this class. (reversed)	.05	*.59*
18b. Time is lost due to child misbehavior. (reversed)	.30	*.53*

*N* = 1358 students in 54 classrooms (24 schools). Factor loadings > .40 are in italics and are included in factor. Extraction method: principal component analysis. Rotation method: Oblimin with Kaiser Normalization

#### Factor variables and correlations

Factor items with loadings above .4 and at least .1 higher than the other factor were used in creating factor variables (i.e., items 7 and 14 were included only in factor one; CLAC items 18a and 18c were not included in either). Instructional responsiveness included items 2, 4, 6–17, 19, and 20; student engagement contained items 1–3, 5, 7, 14, 18b, and 21–23. Factor 1 is highly negatively skewed, with skewness of − .978 with a range of 25–65, *M* = 52.10, *SD* = 7.61. Similarly, factor 2 values range from 29 to 50 (*M* = 42.87, *SD* = 5.17) and its distribution has negative skew, − .57.

The two factors were significantly correlated with one another, *r* (72) = .6743, *p* < .001 as well as with overall task orientation scores, *r* (72) = .76, *p* < .001 and *r* (72) = .68, *p* < .001, respectively.

### Research question 2: what is the predictive validity of the CLAC measure (its overall and two factor scores) to children’s learning using TS-Gold?

Three questions were addressed: to what extent can CLAC’s (a) instructional responsiveness, (b) student engagement, and (c) overall task orientation variables uniquely predict student’s TS-Gold scores, above and beyond a set of potential explanatory variables?

Each CLAC factor, along with covariates, was used as a predictor in linear and probit regression models for the TS-Gold outcome measures: Continuous and dichotomous measures of language, literacy, math, and socio-emotional were evaluated. A total continuous TS-Gold measure was created by compiling subscale scores. A proficiency total score was created by dichotomizing the total subscale score. Using a priori alpha level of .05, CLAC variables with coefficients that fell below this threshold were considered predictors of the student outcomes. Further, effect sizes were used to interpret the strength of these relations.

The models accounted for nesting by clustering the standard error at the classroom level and included the following covariates: baseline performance, assessment date, gender, race, ethnicity, special education status, age, and free lunch eligibility. There are several statistically significant relations between the covariates and TS-Gold outcomes (see Table 7). The dichotomized baseline achievement scores often perfectly predicted proficient outcome measures; consequently, the continuous baseline measures were used in the models. Multiple imputation via the expectation-maximization algorithm was used to fill in missing baseline and end-of-year scores after verification that values were missing at random. This approach increased power and efficiency in estimation.

**Table 7:** Correlation matrix of covariates and spring outcome scores

	1	2	3	4	5	6	7	8	9	10	11
1. Gender	–										
2. Race	− .01	–									
3. Hispanic	.01	*− .91*	–								
4. Special ed	*− .17*	*− .14*	*.12*	–							
5. Age	.001	*− .06*	*.08*	− .001	–						
6. Free lunch	.04	*.19*	*− .15*	− .04	*.07*	–					
7. Assess date	*.04*	*− .11*	*.11*	− .01	*.10*	− .001	–				
8. Language	*.12*	*.07*	*− .07*	*− .27*	*.62*	.001	*.16*	–			
9. Literacy	*.08*	*.12*	*− .11*	*− .20*	*.65*	.02	*.15*	*.87*	**–**		
10. Math	*.07*	*.07*	*− .07*	*− .22*	*.65*	− .02	*.17*	*.87*	*.93*	**–**	
11. Socio-emotional	*.13*	− .03	.03	*− .21*	*.62*	− .004	*.18*	*.90*	*.82*	*.83*	**–**
12. Total Score	*.10*	*.05*	*-.05*	*-.22*	*.67*	.007	*.17*	*.96*	*.95*	*.94*	*.94*

Italicized values indicate significant at *p* < .05

Table 8 presents predictor variables’ model coefficients on each outcome measure along with each models’ effect size and *R*^2^/pseudo *R*^2^. Across all models, the total amount of variance accounted for in the models was quite high for the measures using the continuous predictors: Between 71 and 77% of the variance were accounted for by the individual models, i.e., *R*^2^ = .708, *p* < .001, *R*^2^ = .770, *p* < .001. See Appendices 11, 12, and 13 for complete regression models with covariates for overall task orientation, instructional responsiveness factor, and student engagement factor, respectively.

**Table 8:** Marginal effects and effect sizes from probit and linear regression model predicting year-end learning with CLAC scores

	Math				Literacy				Socio-emotional				Language				Total			
	Continuous		Prof		Continuous		Prof		Continuous		Prof		Continuous		Prof		Continuous		Prof	
	Coef	ES^a^	Coef	ES^b^	Coef	ES^a^	Coef	ES^b^	Coef	ES^a^	Coef	ES^b^	Coef	ES^a^	Coef	ES^b^	Coef	ES^a^	Coef	ES^b^
Factor 1	0.11**	.12	0.02*	.01	0.16*	.09	0.02	.002	0.03	.03	0.01	.002	0.04	.05	0.01	.06	0.39	.07	0.01	.003
Factor 2	0.17**	.10	0.03*	.01	0.30*	.10	0.04*	.01	0.13	.06	0.03^†^	.01	0.11*	.08	0.01^†^	.002	0.87*	.08	0.04*	.01
Overall task orientation	1.53**	.34	0.24^†^	.05	2.47*	.30	0.26	.04	0.39	.07	0.07	.02	0.62	.18	0.08	.01	5.97^†^	.21	0.13	.03

*n* = 1358. Coefficients are marginal (unstandardized) reflecting the change in TS-Gold assessment scores per 1-unit change in the factor score. Model covariates include gender, race, ethnicity, special education status, age, free lunch eligibility, and respective baseline learning. Standard error adjusted for classroom-level clusters (52). In all models, total *R*^2^/pseudo-*R*^2^ ranged from .708–.772 (*R*^2^) to .344–.488 (pseudo-*R*^2^)

*p < .05; **p < .01; ^†^*p* < .1

^a^Effect size (ES) calculation: task orientation is a 2-point change on 5-point scale; factor score changes are calculated as a 1 *SD* change: 10 and 5.25 points for factor 1 and factor 2, respectively

^b^Marginal effects at the mean

#### Overall task orientation

**Fig. 1:**
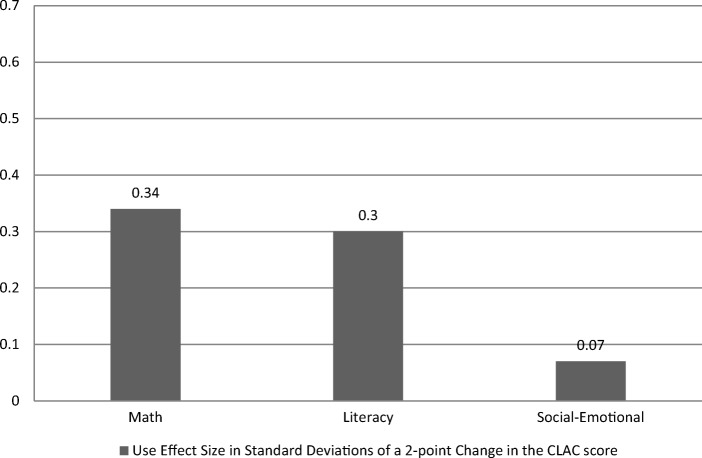
Effect sizes of overall task orientation on end-of-PreK learning. Scores are from the teaching strategies Gold assessment system and account for baseline differences in performance. Task orientation is the combined summary score for the CLAC and includes the instructional responsiveness and student engagement factors

After controlling for potential confounds and nesting effects within classrooms, classroom levels of task orientation predicted the continuous measures of math, *β* = 1.51, *p* < .01 and literacy learning, *β* = 2.42, *p* < .05. To better understand its practice significance, effect sizes were calculated. As seen in Table 8, the effect sizes across the CLAC predictors on student learning ranged from .002 to .34. See Fig. 1 for effect sizes of overall task orientation on student learning at the end of PreK.

#### Instructional responsiveness

As seen in Table 8, classroom levels of instructional responsiveness (factor 1) significantly predicted later continuous and dichotomized math scores, *β* = .11, *p* < .01 and *β* = .02, *p* < 05, respectively. Classroom instructional responsiveness also predicted students’ year-end literacy learning, *β* = .06, *p* < .05 and approached significance in the socio-emotional proficiency regression model, *β* = .01, *p* < .06.

#### Student engagement

The second CLAC factor, student engagement, was a significant predictor in continuous and proficient math learning, *β* = .18, *p* < .01 and *β* = .04, *p* < .05, respectively. Similarly, levels of the student engagement variable significantly predicted literacy learning and year-end proficiency, *β* = .31, *p* < .05 and *β* = .04, *p* < .05, respectively. As seen in Table 8, factor 2 also predicted language scores, *β* = .13, *p* < .05 and both total TS-Gold scores, *β* = .94, *p* < .05 and *β* = .04, *p* < .05, respectively.

To assess the value added of the CLAC, separate regression models were run with each of the CLASS domain scores (emotional support, classroom organization, and instructional support), CLAC scores, and the covariates. While none of the CLASS domains predicted year-end gains, each of the original statistically significant CLAC variables continued to predict student learning. For example, student engagement (factor 2) continued to predict the math learning after including the CLASS domain score: emotional support (*β* = .20, *p* < .01), classroom organization (*β* = .20, *p* < .001), and instructional support (*β* = .19, *p* < .01).

## Discussion

The purpose of this study was to assess for the first time the utility of the Classroom Learning Activities Checklist (CLAC) in measuring classroom task orientation and engagement in learning. The dimensionality and predictive validity were the main foci. The first research question focused on dimensionality. In analyzing the items associated with both factors, two subconstructs emerged: items that reflect classroom supports (instructional responsiveness, associated with factor 1) and observed reception to classroom activities (student engagement, associated with factor 2). The two CLAC factors reinforce existing literature that indicates different kinds of interactions (e.g., individualized attention and child engaging strategies) promote learning for young children (e.g., Burchinal et al. 2008; NICHD Early Child Care Research Network 2002). Specifically, responsive instruction with individualized support for children with low levels of self-regulation is associated with greater self-regulation gains (Connor et al. 2010). Student engagement, as previously examined in the literature review has also been linked to self-regulation (Fantuzzo et al. 2004; Williford et al. 2013). Additionally, these independent yet correlated CLAC factors support the bidirectional relation students have with their teachers and environments. Finally, our 2-factor structure is conceptually consistent with the CLASS domains of emotional support, classroom organization, and instructional support. Although we did not identify a third dimension, the CLAC tool was developed to assess a narrower range of classroom experiences and interactions consistent with the MCPC intervention theory.

The second research question examined CLAC’s predictive validity on children’s learning at the end of the PreK year. Without evidence of the measure’s ability to connect to child-level outcomes, our ability to further explore its potential refinement, scalability, and broader dissemination is limited. After controlling for covariates, each of the three predictors, overall task orientation (CLAC26), factor 1 (instructional responsiveness), and factor 2 (student engagement) significantly predicted year-end learning. The student engagement factor appeared to be the best predictor of children’s learning: seven of the 10 models with factor 2 were associated with significantly higher TS-Gold scores.

While it was encouraging to see evidence of the CLAC’s relation to aspects of student’s math and literacy learning, it was unexpected to *not* see differential impacts of the CLAC factor scores. Further predictive validity research is warranted: these analyses included data from one large Midwestern district using a single assessment, TS-Gold. The validity evidence of these findings would be bolstered by examining differing subpopulations and, more importantly, using additional standardized assessment tools with demonstrated validity.

While there was variability across the items, subscales, and factors, of concern is the consistent restricted response range of the individual CLAC items. After this observation round was conducted in the PreK year and its data analyzed, a number of measures were taken to address the lack of variability. First, a 7-point scale was piloted both independently and in conjunction with the 5-point scale. Observers reported anecdotally that the 7-point measure provided more variation in ratings. The scoring rubric was also revisited, and clarifying text was added where inconsistencies existed. These measures were taken to better distinguish values while maintaining the same scoring schema and subsequent score interpretations across tool versions. The changes were designed to better detect true differences that inherently lie within classrooms that the first CLAC tool was potentially unable to measure.

Finally, the CLAC training process has been continually improved. Annual training is provided for observers where extra time is set aside for in-depth conversations on the operationalized definitions, scoring consistencies, and observing scenarios. Another revision employed is the randomization of the observers. Due to the logistics of collecting classroom observation data within a large-scale, multi-state intervention, on-site support staff often conducted the CLAC observations. While fully trained on the CLAC, we cannot know if the observers were unbiased and rule out a “halo effect” in their scoring.

Future research should examine the circumstances in which scores change across times of day, content focuses, and groupings and, relatedly, the generalizability of the scores. The CLAC observations were scheduled in advance with directions that any instructional activity was observable. Most classrooms (77%) included whole group instruction and only 3% of CLAC observations included routines. Connecting to other research, children often spend much less time in whole group instruction (23%) and substantially more time in routines (35%) (Early et al. 2010).

Moderator analyses should also be investigated. It is plausible, and even likely instructional groupings (whole group, free play, small group) affect student task-oriented learning. Higher levels of children’s engagement are associated with activity settings that allow a greater degree of choice, such as free choice (Vitiello et al. 2012). Similarly, certain content areas may more readily lend themselves to behaviors and instructional supports that foster engaged and active participation. The proportion of teacher-directed and child-initiated instruction may also moderate the relation between classroom task orientation and children’s learning. Finally, further review into the length of observation is recommended.

Moving forward, the CLAC has the potential to effectively guide and shape the classroom strategies and practices that promote student task orientation. While more evidence is needed to support the CLAC as a measure of classroom task orientation, evidence presented here suggests the CLAC connects to aspects of classroom quality, specifically the role of teachers in implementing effective practices.

Supporting task-oriented learning relies heavily on what classroom teachers believe, know, and ultimately do. Individual CLAC items that loaded onto the first factor, instructional responsiveness, often were observed measures of teachers’ direct and indirect teaching interactions and methods. Similarly, student engagement (factor 2) could be interpreted as the result of strategies teachers have employed that promote positive behavior management and classroom engagement. While evidence of the professional development (PD) interventions’ impact on task orientation is unknown, changes in specific teacher interactions have been observed across different learning domains, including language and literacy (McCollum et al. 2013; Piasta et al. 2012; Powell et al. 2010) and social-emotional interactions (Hamre et al. 2012; Hemmeter et al., 2011; Raver et al. 2008). Further, the CLAC may be a valuable tool in providing data to inform a variety of classroom and programming interests. The CLAC may serve to inform broader program quality via progress monitoring or more summative evaluation. Additionally, the CLAC could potentially be used to assess the impact of specific interventions (e.g., those that target student engagement). Regardless of its application, it is imperative data from CLAC observations directly inform the very practices it measures.

In conclusion, findings show that the CLAC measures two dimensions of classroom context—instructional responsiveness and student engagement. Each dimension was independently associated with PreK learning gains. Findings enhance understanding of how effective classroom strategies and environments affect student learning through the development of self-regulation and task orientation. Facilitating students’ early self-control and self-directed behavior provides a strong foundation for learning (Fitzpatrick and Pagani 2013) that helps ensure that gains can be sustained as children transition to kindergarten and the elementary grades.

## Appendix

**Table 9:** Classroom Learning Activities Checklist items 1–23, 26

1. Children appear fully engaged in activities.	
2. Children are active participants in their learning.	
3. Children appear to be working/oriented towards a goal/learning objective.	
4. Children are engaged with peers and/or materials.	
5. Children’s attention to the lesson is evident.	
6. Child sharing of answers and thoughts is frequently observed.	
7. Organization of lesson and materials are conducive to task orientation.	
8. Teaching methods promote engagement.	
9. Teaching methods facilitate active participation.	
10. Teacher shows openness/responsiveness to active participation and student engagement.	
11. Individual attention to children is evident.	
12. Extra help is provided to children when needed.	
13. Responsiveness to children’s work and behavior is frequent.	
14. Activities provided consistently engage children.	
15. Activities support active participation.	
16. A variety of activities are provided.	
17. There is a blend of teacher-directed and child-initiated activities.	
18 (a). Learning time was lost due to lack of teacher preparedness.	
18 (b). Learning time was lost due to student misbehavior.	
18 (c). Learning time was lost due to noninstructional time/routines.	
19. The pace of activities matches children’s interests and attention.	
20. The amount of time in the lessons/activities matches children’s interests and attention.	
21. Child misbehavior is a problem in this class.	
22. Children follow rules and directions.	
23. Children demonstrate positive peer relations.	
26. Overall task orientation.	

Each item was coded 1–5 (1 = strongly disagree/never/none; 2 = disagree/rarely/few; 3 = neutral/sometimes/some; 4 = agree/most of the time/many; 5 = strongly agree/always/nearly all)

**Table 10:** Classroom Learning Activities Checklist (CLAC) Principal Components Varimax Component Matrix

	Component	
	1	2
15. Activities support active participation.	*.86*	.12
8. Teaching methods promote engagement.	*.80*	.18
16. A variety of activities are provided.	*.79*	.07
9. Teaching methods facilitate active participation.	*.77*	.19
10. Teacher shows openness/responsiveness to active part. and engagement.	*.69*	.31
20. The amount of time in the lessons/activities matches children’s interests.	*.68*	.21
6. Child sharing of answers and thoughts is frequently observed.	*.66*	.17
13. Responsiveness to children’s work and behavior is frequent.	*.65*	.10
17. There is a blend of teacher- directed and child- initiated activities.	*.64*	.01
14. Activities provided consistently engage children.	*.63*	*.45*
11. Individual attention to children is evident.	*.60*	.04
12. Extra help is provided to children when needed.	*.59*	-.04
4. Children are engaged with peers and/or materials.	*.56*	.39
2.Children are active participants in their learning	*.55*	*.45*
7. Organization of lesson and materials are conducive to task orientation.	*.48*	*.44*
18a. Time is lost to lack of teacher preparation. (reversed)	.28	.14
5. Children’s attention to the lesson is evident.	.26	*.81*
1. Children appear fully engaged in activities.	.31	*.78*
3. Children appear to be working/oriented towards a goal/learning objective.	.23	*.76*
23. Children demonstrate positive peer relations	.02	*.75*
22. Children follow rules and directions.	-.14	*.75*
21. Misbehavior is a problem in this class. (reversed)	.11	*.69*
18b. Time is lost due to child misbehavior. (reversed)	.33	*.61*
18c. Time is lost due to routines. (reversed)	.01	.05

Factor loadings > .40 are in italics and are included in factor. Extraction method: principal component analysis. Rotation method: Varimax with Kaiser Normalization

**Table 11:** Linear and probit regression models predicting student outcomes with overall task orientation

	Language		Literacy		Math		Socio-emotional		Total	
	Cont.	Prof.	Cont.	Prof.	Cont.	Prof.	Cont.	Prof.	Cont.	Prof.
CLAC26	0.62	0.08	2.47*	0.26	1.53**	0.24	0.39	0.07	5.97	0.13
Baseline	0.70***	0.22***	0.74***	0.10***	0.71***	0.16***	0.63***	0.09***	0.71***	0.03***
Baseline date	1.84**	0.69***	1.91	0.56**	1.45	0.42*	2.20	0.50*	11.62*	0.80***
Gender	0.28	0.14	0.34	− 0.15	− 0.44	− 0.21*	0.71**	0.21**	0.76	0.003
Race	0.53	0.13	0.44	0.01	− 0.25	0.20	− 0.48	− 0.08	0.26	0.02
Hispanic	− 0.65	0.05	− 1.72	− 0.10	− 1.15	− 0.15	− 0.99	− 0.14	− 6.60	− 0.05
Special education	− 1.06	− 0.72**	− 4.53***	− 0.65***	− 3.50***	− 0.87***	− 2.36*	− 0.56**	− 13.05**	− 0.89***
Age (months)	− 0.09	− 0.08***	0.38**	− 0.09***	0.25**	− 0.08***	0.19	− 0.04	1.12*	− 0.09***
Free lunch eligible	− 0.65	− 0.50**	0.30	− 0.43**	− 0.36	− 0.60**	− 0.06	− 0.22	− 0.60	− 0.31
*R*^2^/pseudo-*R*^2^	.484	.484	.717	.385	.735	.356	.717	.321	.779	.427

Standard error adjusted for 52 classroom-level clusters

**p* < .05; ***p* < .01; ****p* < .001

**Table 12:** Linear and probit regression models predicting student outcomes with CLAC factor 1, instructional responsiveness

	Language		Literacy		Math		Socio-emotional		Total	
	Cont.	Prof.	Cont.	Prof.	Cont.	Prof.	Cont.	Prof.	Cont.	Prof.
Factor 1	0.04	0.01	0.16*	0.02	0.11**	0.02*	0.03	0.01	0.39	0.01
Baseline	0.70***	0.22***	0.75***	0.11***	0.72***	0.16***	0.63***	0.09***	0.72***	0.03***
Baseline date	1.81**	0.68**	1.77	0.55**	1.34	0.40*	2.18	0.50*	11.25*	0.80***
Gender	0.30*	− 0.14	0.41	− 0.13	− 0.39	− 0.20*	0.72**	0.21***	0.91	0.004
Race	0.60	0.15	0.73	0.03	− 0.05	0.23	− 0.42	− 0.07	1.02	0.04
Hispanic	− 0.86	0.004	− 2.57*	− 0.21	− 1.70	− 0.25	− 1.14	− 0.17	− 8.69	− 0.11
Special education	− 1.03	− 0.71**	− 4.53***	− 0.65***	− 3.47***	− 0.86***	− 2.35*	− 0.55**	− 12.84**	− 0.88***
Age (months)	0.09	− 0.08***	0.38**	− 0.09***	0.25***	− 0.08***	0.19	− 0.04**	1.11*	− 0.09***
Free lunch eligible	− 0.64	− 0.51**	0.25	− 0.44**	− 0.39	− 0.62***	− 0.07	− 0.22	− 0.73	− 0.32
*R*^2^/pseudo-*R*^2^	.778	.485	.716	.383	.740	.361	.717	.322	.779	.429

Standard error adjusted for 52 classroom-level clusters

**p* < .05; ***p* < .01; ****p* < .001

**Table 13:** Linear and probit regression models predicting student outcomes with CLAC factor 2, student engagement

	Language		Literacy		Math		Socio-emotional		Total	
	Cont.	Prof.	Cont.	Prof.	Cont.	Prof.	Cont.	Prof.	Cont.	Prof.
Factor 2	0.11*	0.03	0.30*	0.04	0.17**	0.03*	0.13	0.03	0.87*	0.04*
Baseline	0.69***	0.22***	0.73***	0.10***	0.70***	0.15***	0.62***	0.09***	0.70***	0.03***
Baseline date	1.72**	0.66***	1.61	0.53**	1.27	0.40*	2.09	0.47*	10.79*	0.78***
Gender	0.30*	0.14	0.39	− 0.13	− 0.41	− 0.20*	0.73**	0.22***	0.89	0.01
Race	0.70	0.18	0.93	0.04	0.02	0.24	− 0.32	− 0.05	1.55	0.06
Hispanic	− 0.67	0.06	− 1.97	− 0.15	− 1.32	− 0.18	− 0.97	− 0.12	− 7.07	− 0.04
Special education	− 1.00	− 0.71**	− 4.34***	− 0.63***	− 3.39***	− 0.85***	− 2.29*	− 0.54**	− 12.64**	− 0.88***
Age (months)	0.11	− 0.07***	0.42**	− 0.09***	0.28***	− 0.08***	0.20	− 0.04	1.24*	− 0.08***
Free lunch eligible	− 0.54	− 0.49**	0.61	− 0.40**	− 0.18	− 0.56**	0.08	− 0.19	0.30	− 0.29
*R*^2^/pseudo-*R*^2^	.782	.488	.716	.387	.735	.358	.720	.330.	.780	.435

Standard error adjusted for 52 classroom-level clusters

**p* < .05; ***p* < .01; ****p* < .001
